# Mitral valve repair in fibroelastic deficiency

**DOI:** 10.1186/1749-8090-10-S1-A44

**Published:** 2015-12-16

**Authors:** Gregorio P Cuerpo, Carlos A Rivera, Diego F Sánchez, S Costanza, Angel G Pinto

**Affiliations:** 1 Department of Cardiac Surgery Hospital Gregorio Marañón, Madrid, Spain, 28032

## Background/Introduction

Fibroelastic deficiency (FED) in the mitral valve might be challenging in certain patients. Leaflets are thin and ruptured chordae are often present. The number of techniques to be applied might be numerous and compromise long-termr esults

## Aims/Objectives

To evaluate long-term results in the setting of mitral valve repair (MVR) for fibroelastic deficiency since our shift towards non-resective techniques from Carpentier's classical repair options

## Method

Single-institutional retrospective study with echocardiographical and clinical follow-up performed between 2005 and 2014. End-points observed were mortality and freedom from mitral insufficiency >II/IV.

## Results

158 patients were considered to have FED and had MVR in our institution from 2005 to 2014. Mean follow-up was 1468 days. Mean age was 65,8 years. Preoperative functional status (FS) was NYHA III-IV in 61 patients (38,6%).

There were 4 in-hospital deaths (4/158) none of them in the operating room (OR) 6 out of 158 left the OR with >2+ mitral insufficiency (MI).

At discharge there were 15 deaths. Actuarial survival was 89,6% (13 deaths/154)

Freedom from >2+ MI was 92,8% (129/139) with 6 patients being reoperated during follow-up (4 underwent MV replacement and 2 cardiac transplantation).

Freedom from FS >III+ was 132/139.

No endocarditis were observed during follow-up

## Discussion/Conclusion

Long-term results for FED mitral valve repair seem to be excellent being apparently the primary option for these patients. Endocarditis and thromboembolic events appear to be almost forgotten in this setting.

